# Supplementary material for: Design and Validation of the INCUE Questionnaire: Assessment of Primary Healthcare Nurses’ Basic Training Needs in Palliative Care
Source: Int J Environ Res Public Health. 2021 Oct 19;18(20):10995. doi: 10.3390/ijerph182010995 (PMC8535240; doi:10.3390/ijerph182010995)
Supplement: Supplementary file 1 [file ijerph-18-10995-s001.zip › ijerph-1398475-Supplementary material.pdf]

Table S1. The INCUE questionnaire to assess primary and home healthcare nurses' basic training needs in palliative care.

Part 1		Part 2	
Based on your knowledge of Palliative Care, answer the following questions:	*Response options*	*Answer the following questions based on your clinical practice:*	*Response options*
Area 1: Principles of palliative care			
1. According to the philosophy of Palliative Care, the patient and relatives form the unit to be treated.<br>2. Palliative Care considers death as a natural process.<br>3. Palliative Care should be provided only to people with a life expectancy of less than 6 months.<br>4. Palliative Care is performed only by Palliative Care Units.	Yes<br>No<br>Do not know / no answer	24. Do you work as part of a team in your healthcare activity?<br>25. Do you assess the needs of family members?<br>26. Do you intervene in the needs of family members?<br>27. Do you use an instrument to identify patients with palliative needs?<br>28. Do you provide spiritual guidance as part of the care of the palliative patients you attend to?<br>29.Do you consider the quality of life of palliative patients?	Never<br>Rarely<br>Sometimes<br>Often<br>Always
Area 2: Symptom management and specific care plans			
5. The visual analog scale (VAS) scale is used to measure pain intensity.<br>6. Oral administration of morphine is ineffective.<br>7. Pain is the same as suffering.<br>8. Palliative sedation is indicated when there are one or more refractory symptoms.<br>9. The NECPAL is an instrument used to identify people with palliative care needs.	Yes<br>No<br>Do not know / no answer	30. Do you use some sort of assessment scale in your daily work?<br>31. Do you perform or teach relatives mouth-care of dependent patients, with palliative sedation or short-term life prognosis?<br>32. After home administration of a prescribed pain control drug, do you assess its effectiveness?<br>33. Do you teach relatives how to prepare and administer medication subcutaneously?<br>34. Do you employ non-pharmacological measures to assist in symptom control?<br>35. Do you perform periodic follow-up according to the needs of people in palliative situation?	Never<br>Rarely<br>Sometimes<br>Often<br>Always
Area 3: Coping with loss and death			
10. Anger is one of the phases of adapting to loss.<br>11. Palliative Care ends with the death of the patient.<br>12. Patients may experience grief.<br>13. Complicated grief issues require referral to a Mental Health Unit.<br>14. There are scales to rate risk factors associated to complicated grief.	Yes<br>No<br>Do not know / no answer	36. Do you identify the phases of coping with the disease of a palliative patient?<br>37. Do you encourage relatives express their emotions after the death of the patient?<br>38. Do you carry out periodic follow-up of the relatives after the patient's death?<br>39. Do you evaluate the mourner's support network?<br>40. Do you use instruments to assess the risk of complicated grief?<br>41. Do you refer people you consider at risk of complicated grief to psychology / psychiatry units?	Never<br>Rarely<br>Sometimes<br>Often<br>Always
Area 4: Communication skills			
15. When listening, we should keep an upright posture with our arms folded.<br>16. For suitable communication, we should remain at the foot of the patient's bed.<br>17. Patient suffering can produce compassion fatigue in professionals.<br>18. During active listening, you should offer simple solutions, talk about yourself and minimize feelings.	Yes<br>No<br>Do not know / no answer	42. Do you intervene in situations where the patient expresses discomfort or anger?<br>43. Do you explain the care to be administered to patients previous to their entering a state of palliative sedation or decreased level of consciousness?<br>44. Do you pay attention to the patient's nonverbal language in your daily work?<br>45. Do you place yourself at the same height as the patient when conducting the interview for evaluation?<br>46. Do you explore the patient's concerns and feelings?<br>47. Do you identify the needs of relatives?	Never<br>Rarely<br>Sometimes<br>Often<br>Always
Area 5: Ethical and legal issues			
19. We must respect the patient's wishes, even though we consider there are better therapeutic options for him/her.<br>20. The wishes of the patient must prevail over those of their relatives.<br>21. There is a national register of advance directives or living will.<br>22. Advance directives may be revoked.<br>23. Sedation may be applied to a patient in full use of his/her mental faculties without his/her consent in the last days of life.	Yes<br>No<br>Do not know / no answer	48. Do you tailor patient care to their preferences?<br>49. Do you involve the patient and their relatives in decision-making?<br>50. Do you inform palliative patients that there is an advance directives or living will?<br>51. Do you respect the patient's decisions, even if they are considered inappropriate?<br>52. Do you participate in decision-making?<br>53. Do you take into account the cultural characteristics of the person and/or family, when administering care?	Never<br>Rarely<br>Sometimes<br>Often<br>Always
